# A Comparative Study for Nutritional and Phytochemical Profiling of *Coffea arabica* (*C. arabica*) from Different Origins and Their Antioxidant Potential and Molecular Docking

**DOI:** 10.3390/molecules27165126

**Published:** 2022-08-11

**Authors:** Akhtar Ali, Hafza Fasiha Zahid, Jeremy J. Cottrell, Frank R. Dunshea

**Affiliations:** 1 School of Agriculture and Food, Faculty of Veterinary and Agricultural Sciences, The University of Melbourne Australia, Parkville, VIC 3010, Australia; 2 Faculty of Biological Sciences, The University of Leeds, Leeds LS2 9JT, UK

**Keywords:** coffee, polyphenols, antioxidants, human health, molecular docking, LC-MS/MS

## Abstract

Coffee is the most widely used beverage globally and contains many bioactive compounds, including phenolic compounds, alkaloids, triterpenes, organic acids, amino acids, hormones, and fatty acids. The main objective of this study was the comparative profiling of Australian, Colombian, Ethiopian, and Peruvian *C. arabica* using LC-ESI-QTOF-MS/MS. In this study, we tentatively identified 136 bioactive metabolites, including five (05) organic acids, six (06) alkaloids, three (03) amino acids (l-phenylalanine, l-tyrosine, and l-pyroglutamic acid), two (02) hormones (melatonin and serotonin), two fatty acids, one (01) furopyrans (goniothalenol), one (01) carotenoid (crocetin), three (03) terpenoids, thirty-eight (38) phenolic acids, forty-one (41) flavonoids, five (05) stilbenes, three (03) lignans and twenty-three (23) other polyphenols in *C. arabica*. The highest TPC value (17.74 ± 0.32 mg GAE/g) was measured in Colombian coffee while the lowest TPC value (10.24 ± 0.73 mg GAE/g) was in Peruvian coffee. Colombian coffee has a higher antioxidant potential than other studied coffee samples. A total of nineteen phenolic metabolites were mapped through LC-MS/MS. Quinic acid derivatives were quantified in higher concentrations than other metabolites. Furthermore, molecular docking predicted that chlorogenic acid is a main bioactive compound that contributes to anti-Alzheimer and anti-diabetic activities of *C. arabica*. The obtained results indicate that *C. arabica* contains a vast number of bioactive compounds which have potential health benefits. Furthermore, research could be conducted to validate the effect of these metabolites on the flavor profile of coffee beverages.

## 1. Introduction

Coffee is one of the main agricultural products consumed all over the world, mostly in developed countries. The major types of coffees differ based on the processing or roasting conditions or methods, which affect the chemical composition of the final product and are known as brewed, filtered and un-filtered coffee, instant coffee, expresso, boiled and infused coffee, and caffeinated and decaffeinated coffee [1]. There are two major coffee species consumed worldwide: *Coffea arabica* (Arabica) and *Coffea canephora* (robusta). *C. arabica* is one of the most widely cultivated coffees, consumed in the world, and of superior quality due to its organoleptic properties. Almost 70% of all coffee in the world is *C. arabica* [2].

Coffee is well-known due to various health effects [3] attributed to phytochemicals, including polyphenols, alkaloids, hormones and sesquiterpenoids, and amino acids. Coffee contains nicotinic acid, tannic acid, and, of course, caffeine. The most abundant bioactive metabolites are caffeine, quinic acid derivatives (chlorogenic acid), caffeic acid, ferulic acid, *p*-coumaric acid, melatonin, and serotonin [4]. Another important class of compounds is melanoidins, which are produced as a result of the Maillard reaction during roasting [5]. Moreover, coffee also contains cinnamic acids, cinnamaldehydes, and proanthocyanidins, which have antioxidant, anti-inflammatory [6], antidiabetic, anticancer [7], cardio-protective [8] and antimicrobial properties [7,9]. The antioxidant activity of coffee is mainly attributed to phenolic compounds due to their ability to scavenge free radicals in the biological system [1]. It also contains abundant phenolic compounds, including quercetin, vanillic acid, *p*-hydroxybenzoic acid, and other bioactive compounds. These compounds in coffee samples act as radical scavengers and are beneficial for human health [1]. Chlorogenic acid most abundant phenolic compound in coffee after caffeine which has antioxidant, anti-cancer, neuro-protective, anti-inflammatory, anti-viral properties [3]. Regardless of some controversial impacts of coffee on health, it is the most widely used beverage worldwide after tea. Coffee is famous for its positive health effects, including antidiabetic potential, high metabolic rate, and the treatment of various metabolic disorders [1,10]. Redox imbalance leads to a biological state called oxidative stress, which leads to the destruction of cellular cells, including DNA [11]. Aging is also a significant factor in producing reactive oxygen species (ROS) in the body, the leading cause of redox imbalance. Therefore, including antioxidants in diets is necessary to fight against diseases [6,12]. Reportedly, coffee has many health benefits including antidiabetic, anti-Alzheimer potential and anti-cancer properties [6,7]. Although some studies reported positive effects of caffeine [7,13], an excessive amount of caffeine can trigger the state of anxiety and excitement including some adverse reactions such as headaches, restlessness, insomnia, nausea, migraines, tremors, tachycardia and elevated blood pressure [7,13,14]. Young children, pregnant women, developing fetuses and those with liver disease are the susceptible groups to the toxicity of caffeine consumption [13]. The prediction of the best possible role of small molecules in the inhibition of various enzymes that are the leading cause of target pathologies is important. Molecular docking is the virtual method to study complex biological and chemical systems and predict the role of novel compounds of therapeutic interest [15]. Since 1980, molecular docking has been widely used in drug discovery and is the most common computational structure-based drug design method [16].

There are thousands of phytochemical metabolites in coffee which are not fully characterized yet [1]. This study aimed to explore the complex nutritional and phytochemical profile of *C. arabica* of Australian, Colombian, Ethiopian and Peruvian origins by using LC-ESI-QTOF-MS/MS. Furthermore, total phenolic content (TPC), total flavonoid content (TFC), and their in vitro antioxidant activities were also examined in this study. Additionally, in vitro antioxidant assays 2,2′-diphenyl-1-picrylhydrazyl (DPPH), 2,2′-azino-bis-3-ethylbenzenzothiazoline-6-sulfonic acid (ABTS) and hydroxy-radical scavenging activity (^•^OH-RSA) were conducted to understand the total antioxidant potential of *C. arabica* of different origins. Furthermore, molecular docking was conducted to predict the role of abundant bioactive compounds in the inhibition of acetylcholinesterase (AChE) and α-glucosidase enzymes to understand the role of coffee metabolites in anti-diabetic and anti-Alzheimer activities.

## 2. Results and Discussion

### 2.1. Measurement of Phenolic Compounds and Antioxidant Potential of C. arabica

Phenolic compounds are potent bioactive compounds with strong antioxidant, anti-inflammatory, and anti-carcinogenic activities. Coffee is a rich source of phenolic compounds. Phenolic compounds were estimated through total phenolic content (TPC), total flavonoid content (TFC), and total condensed tannin (TCT). The results for the estimation of phenolic contents are presented in Table 1.

It is believed that TPC represents all types of phenolic compounds. The Folin–Ciocalteu (F–C) method is the most widely used method for measuring the phenolic contents in plant extracts. The highest TPC value (17.74 ± 0.32 mg GAE/g) was measured in Colombian coffee while the lowest TPC value (10.24 ± 0.73 mg GAE/g) was measured in Peruvian coffee. Overall, the TPC was quantified in the range of 10 to 18 mg GAE/g). Previously, Król et al. [17] quantified total phenolic content in the range of 7.95–8.74 mg/g in organic roasted and non-roasted coffee. The TPC of *C. arabica* was higher than tamarind (3.72 ± 0.12 mg GAE/g) and mountain pepper (5.91 ± 0.32 mg GAE/g), as reported by Cáceres-Vélez et al. [18]. The TPC value of *C. arabica* was higher than a previously reported value of fennel (8.31 ± 0.03 mg GAE/g) and fenugreek (7.58 ± 0.35 mg GAE/g) [19,20]. Roasting is an important step in coffee making, affecting the phenolic contents [21]. There is a huge difference in phenolic contents in different coffee beans due to different roasting conditions, cultivars, and geographical locations [22]. Different extraction methods also affect the total phenolic contents of coffee beans. The TPC value might be different due to different cultivars being used in the current and latter studies for the extraction and quantification of phenolic contents [19]. Overall, coffee is a rich source of antioxidant phenolic constituents. Furthermore, the other major phenolic compounds in coffee beans are flavonoids. The highest TFC value (1.36 ± 0.05) was measured in Colombian coffee while the lowest TFC value (1.01 ± 0.13 mg QE/g) in Peruvian coffee (Table 1). Previously, Wu, Lu, Liu, Sharifi-Rad and Suleria [21] measured TFC in the range of 0.87 to 1.16 in light to dark roasting coffee samples.

Antioxidant activity is the ability of bioactive compounds to protect biological systems against oxidative stress. In this study, we conducted different assays to map and understand the antioxidant potential of *C. arabica*. Bioactive phenolic compounds contribute to the antioxidant potential in coffee. The antioxidant potential is a parameter for nutritionists and dietitians to measure the positive impact on human health [20]. Phenolic compounds act as metal chelators, hydrogen donating compounds, and anti-radicals in the biological system. There is a range of bioactive compounds in coffee beans that act as therapeutic agents to scavenge free radicals in the body such as alkaloids, flavonoids, phenolic acids, coumarins, lignans, stilbenes, tannins, and other polyphenols [23]. DPPH is the inexpensive and cost-effective assay widely used to estimate the antioxidant ability of plant extracts. In this study, the highest DPPH value (3.61 ± 0.06 mg AAE/g) was quantified in Colombian coffee while the lowest DPPH value (1.55 ± 0.03 mg AAE/g) was measured in Peruvian coffee. The ^•^OH-RSA (hydroxyl ion–radical scavenging assay) is the leading assay that help to understand the mechanism of lipid peroxidation because ^•^OH radicals cause DNA damage while coffee extract helps to scavenge the free ^•^OH radicals [19]. The highest ABTS (17.17 ± 0.15 mg AAE/g) and ^•^OH-RSA (23.62 ± 0.47 mg AAE/g) values were measured in Colombian coffee. Overall, Colombian coffee has a higher total phenolic content and antioxidant potential than other selected coffees. Flavonoids may have higher free-radical scavenging capacity due to the presence of the 3′,4′-dihydroxy structure of the ring B in quercetin [24]. Melanoidins that are produced during the roasting may also contribute to antioxidant activity [25]. Melanoidins, heterocyclic compounds and reductive ketones which are produced during roasting also have effective antioxidant capacity [26]. Different antioxidant activities help to understand the targeted antioxidant potential of coffee while LC-MS/MS further elucidate the structures of bioactive compounds that help to build a scientific approach to treat different health problems. Inclusion of different antioxidant bioactives in the daily diet is essential to fighting against different pathological conditions.

### 2.2. Pearson’s Correlation Analysis of Phenolic Contents and Antioxidant Activities

A Pearson correlation analysis was conducted between TPC, TFC and antioxidant activities (Table 2).

It is documented that total polyphenols and total flavonoids are responsible for antioxidant potential [19]. TPC was more correlated with ^•^OH-RSA and ABTS with *r*^2^ = 0.97 (*p* < 0.05) and *r*^2^ = 0.92 (*p* < 0.01) while TFC was more correlated with ABTS (*r*^2^ = 0.99, *p* < 0.05). ABTS also correlated with DPPH and ^•^OH-RSA (*r*^2^ = 0.92, *r*^2^ = 0.94), respectively. Coffee samples contain a number of phytochemical metabolites including flavonoids, phenolic acids, terpenoids and alkaloids, which have strong antioxidant potential [20].

### 2.3. LC-MS/MS Identification of Metabolites from C. arabica

Secondary plant metabolites, including phenolic acids, flavonoids, coumarins, alkaloids, triterpenes and other bioactive metabolites, have associated therapeutic properties [27]. LC-ESI-QTOF-MS/MS is an analytical tool that is widely used for the identification of unknown bioactive compounds. In this study, a total of 136 bioactive metabolites were tentatively identified and further characterized based on their MS/MS spectra (Table S1). Base peak chromatograms (BPC) of Australian, Colombian, Ethiopian and Peruvian coffee in positive and negative modes are given in Figure S1.

#### 2.3.1. Nutritional Profile of *C. arabica*

Organic acids, amino acids and fatty acids are included in the nutritional contents of coffee. In this study, we identified a total of five organic acids (citric acid, malic acid, fumaric acid, mandelic acid and quinic acid), three amino acids (l-phenylalanine, l-tyrosine and l-pyroglutamic acid) and two fatty acids (oleic acid and linoleic acid) in *C. arabica.* Organic acids are low molecular metabolites that contribute to the coffee’s flavour and taste profile [2]. Citric acid and malic acid are widely present in plants. They are considered as energy metabolites in the human body [2]. Previously, Jham et al. [28] and López-Froilán, Ramírez-Moreno, Podio, Pérez-Rodríguez, Cámara, Baroni, Wunderlin and Sánchez-Mata [2] also identified citric acid, malic acid, quinic acid and fumaric acid in coffee. Two essential fatty acids (oleic acid and linoleic acid) were also identified in this study. Fatty acids have been reported for their health benefits [29]. l-Tyrosine (C_9_H_11_NO_3_), l-pyroglutamic acid (C_5_H_7_NO_3_) and l-phenylalanine (C_9_H_11_NO_2_) were identified in positive mode at *m*/*z* 182.0838, 130.0605 and 166.0880, respectively. Furthermore, confirmation was performed through their MS/MS product ions (Table S1). Previously, Dong et al. [30] quantified these amino acids in *Coffea robusta* while Casal et al. [31] also quantified these amino acids in *C. arabica* originating from Brazil. It is evident that amino acids are also potent antioxidant metabolites and have a positive impact on human health [32]. Amino acids act as the building blocks in the body and help to produce the enzymes to keep the body functioning correctly [33]. Previously, l-phenylalanine, l-tyrosine and l-pyroglutamic acid were also identified in coffee by Martins et al. [34].

#### 2.3.2. Hormones

Melatonin (*N*-acetyl-5-methoxytryptamine) and serotonin (5-hydroxytriptamine) are widely studied hormones with potential health effects. In coffee samples, melatonin and serotonin were tentatively identified at *m*/*z* 233 and *m*/*z* 177 in positive modes. Previously, melatonin has been identified in coffee, tea and many other plants in small quantities [35]. Ramakrishna et al. [36] also quantified the melatonin and serotonin in *C. arabica* in the range of 6–9 μg/g and 8–12 μg/g, respectively. Melatonin has been reported to have a role in scavenging free radicals from the biological system [35]. It has been reported that coffee helps to decrease Alzheimer’s disease (AD) and Parkinson’s disease, but the mechanism is not fully elucidated yet [37]. While Corpas et al. [38] reported that it is melatonin that helps to protect against AD, clinical evidence not yet validated.

#### 2.3.3. Alkaloids

**Figure 1:**
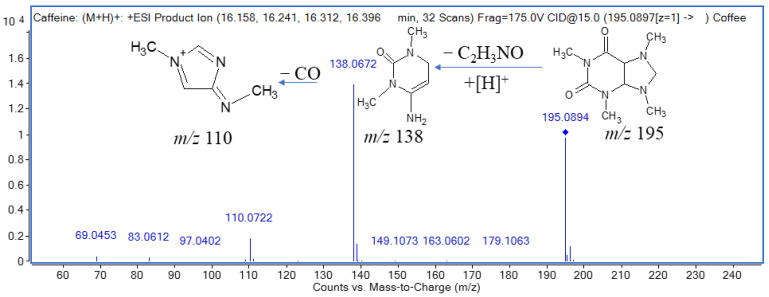
LC-MS/MS identification of caffeine in ESI^+^ mode and proposed fragmentation pattern of caffeine at *m*/*z* 195.

A total of six alkaloids (caffeine, fontanesine B, trigonelline, theophylline, β-carboline and vasicine) were tentatively identified in *C. arabica*. Caffeine (compound **17**) and fontanesine B (compound **16**) were tentatively identified at *m*/*z* 195.0894 and *m*/*z* 370.1541 as alkaloids. Previously, fontanesine B has been reported for its anti-proliferative activities [39]. Vasicine is also an alkaloid found in *C. arabica*. Caffeine belongs to a class of alkaloids naturally present in various plants, including coffee, cocoa and tea. Caffeine has strong antioxidant potential. Previously, 4.01 ± 0.02 to 4.29 ± 0.02 mg/100 g caffeine was estimated in methanol extracts. In this study, caffeine (C_8_H_11_N_4_O_2_) was identified in a positive mode which was further confirmed in MS/MS at *m*/*z* 138 and *m*/*z* 110 after the removal of C_2_H_3_NO from the precursor ion (Figure 1).

#### 2.3.4. Triterpenes

Crocetin (carotenoid) and corosolic acid (triterpene) were also tentatively identified in *C. arabica* in a positive mode. Corosolic acid has antioxidant, anti-inflammatory [40], antidiabetic and anti-cancer properties [41] and has been identified in banana extracts. Crocetin is a well-known constituent of saffron tentatively identified in *C. arabica* and reported for many promoting properties. Kahweol (compound **21**) and cafestol (compound **22**) were also tentatively identified in coffee samples.

#### 2.3.5. Phenolic Acids

Phenolic acids are vital phenolic constituents that have a role in disease prevention due to their strong antioxidant potential. In this study, 38 phenolic acids were identified and characterized with the help of LC-ESI-QTOF-MS/MS in both positive and negative modes (Table S1).

##### Hydroxybenzoic Acid Derivatives

Compounds **25** (protocatechuic acid) and **28** (2-hydroxybenzoic acid) were characterized in a negative mode at *m*/*z* 109 and 93 after with their characteristic loss of CO_2_ from the precursor ion, respectively, confirmed through MS/MS fragmentation pattern [20]. Almost all phenolic compounds lose CO_2_ (44 Da) from the parent ion metabolites [19]. Protocatechuic acid is a unique phenolic acid, widely distributed in medicinal plants [42]. It has been reported for various health properties such as anti-inflammatory, anti-oxidant, anti-ulcer, anti-cancer, and anti-aging activity; anti-microbial, anti-viral, and cardio-protective activity; neuro- and hepatoprotective activity; and anti-diabetic activity, etc. [42]. Syringic acid, *m*-toluic acid and 4-hydroxybenzoic acid 4-*O*-glucoside were also putatively identified in *C. arabica*. Syringic acid has also been reported for its various health benefits [43].

##### Hydroxycinnamic Acid Derivatives

**Figure 2:**
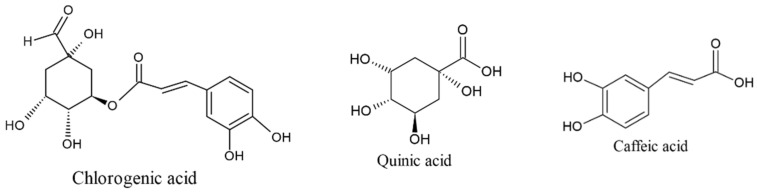
Structures of chlorogenic acid, quinic acid and caffeic acids identified in *C. arabica*.

This is the largest and more diverse group in phenolic acids with significant health potential. In this group, a total of 27 hydroxycinnamic acid derivatives were tentatively identified in *C. arabica*. Compound **32** (3-caffeoyl quinic acid) at *m*/*z* 353 produced fragment ions at 191 (quinic acid) and 179 (caffeic acid) after the removal of glucoside moiety [M−H−162] from the precursor ion while caffeic acid (*m*/*z* 179) further breaks down into *m*/*z* 161 and *m*/*z* 135 after the removal of a water unit (18 Da), and a unit of CO_2_ (44 Da) (Compound **33**, Figure S2). Compounds **41** (*m*/*z* 147) and **52** (*m*/*z* 163) produced fragment ions at *m*/*z* 103 and *m*/*z* 119 after the removal of CO_2_ (44 Da) from the precursor ions, respectively, and were identified as cinnamic acid (C_9_H_8_O_2_) and *p*-coumaric acid (C_9_H_8_O_3_). The structures of chlorogenic acid, quinic acid and caffeic acid are given in Figure 2. Interestingly, rosmarinic acid (compound **35**) was also identified in *C. arabica*, which is present in minute quantities. As per our best knowledge, this is first time we tentatively identified rosmarinic acid in coffee. Compound **29** at ESI^−^ *m*/*z* 325.0937 generated a characteristic product ion of coumaric acid at *m*/*z* 163 after the loss of glucoside moiety [M−H−162] from the precursor ion. Compound **29** was tentatively identified as *p*-coumaric acid 4-*O*-glucoside.

#### 2.3.6. Flavonoids

Flavonoids are the major class of phenolic compounds that contains more than 10,000 metabolites, and these metabolites have vital health effects. A total of 41 flavonoids (five flavanols, eight flavonols, three flavones, three flavanones, fifteen isoflavonoids, one dihydroflavonols and four dihydrochalcones) were tentatively identified in *C. arabica*. Compound **62** at *m*/*z* 479 in negative mode produced fragment ions at *m*/*z* 149 [M−H−C_17_H_15_O_7_]^−^ and *m*/*z* 121 [M−H−C_15_H_18_O_10_]^−^ after the sugar unit and B-ring cleavage from the precursor ion, respectively, which is likely identified as 3′-*O*-methyl-(−)-epicatechin-7-*O*-glucuronide (C_22_H_24_O_12_). Compound **64** at *m*/*z* 289 produced a main fragment ion at *m*/*z* 245 after removing CO_2_ from the precursor ion and was identified as epicatechin (C_15_H_14_O_6_). Catechins are an important class of flavonoids present in coffee and tea [44,45].

#### 2.3.7. Stilbenes and Lignans

Lignans and stilbenes are widely studied secondary metabolites due to their structural diversity and proven positive health effects on human health. In this study, we tentatively identified nine metabolites in these groups. Compound **106** showed a precursor ion at *m*/*z* 243 which is a deprotonated ion of piceatannol which produced a fragment ion at *m*/*z* 225 after the removal of a water unit [M−H−H_2_O] from the precursor ion, while a second product ion was produced at *m*/*z* 201 due to neutral loss C_2_H_2_O (42 Da) from the precursor ion. Piceatannol contains two phenol rings and is most widely studied in red wine and grapes. Previously, piceatannol was identified and semi-quantified in fenugreek and dill leaves. It has been reported to have potent antioxidant, anti-cancer, anti-mutagenic and anti-inflammatory properties [19].

#### 2.3.8. Other Polyphenols

A total of 22 other polyphenols were putatively identified in *C. arabica*. Hydroxytyrosol (compound **128**) was identified at *m*/*z* 153 in negative mode which was further confirmed in MS/MS where it produced product ions at *m*/*z* 123 and 109 after the removal of CH_2_OH and CH_2_-CH_2_-OH from the precursor ion, respectively. Previously, Hamden et al. [46] also reported the same fragmentation pattern of hydroxytyrosol identified in olive. Carnosol (compound **130**) and carnosic acid (compound **131**) were identified in negative mode at *m*/*z* 329 and 331 which produced daughter ions at *m*/*z* 285 and 287 after the removal of CO_2_ [M−H−44 Da] from the precursor ions, respectively. Carnosol and carnosic acid belong to phenolic terpenes. Previously, Wang et al. [47] also reported carnosol and carnosic acid in their study. Phenolic terpenes have been used as antioxidant agents for the prevention of diseases in the pharmaceutical area of research, as reported by Zabot et al. [48]. Compound **135** was identified as pyrogallol at *m*/*z* 125 in a negative mode, which further confirmed in MS/MS mode where it produced a consistent product ion at *m*/*z* 107 after the loss of an H_2_O unit (18 Da) from the precursor ion (Figure S2). Previously, Zhao et al. [49] also identified and confirmed the MS/MS spectra of pyrogallol through UHPLC-QTOF/MS and NMR [49].

The application of LC-ESI-QTOF-MS/MS allowed us to identify 136 bioactive metabolites in *C. arabica*. This will allow us to confirm the different biological activities based on these bioactive metabolites at a fractional level after purification of these compounds.

### 2.4. Distribution of Nutritional and Phytochemical Metabolites

**Figure 3:**
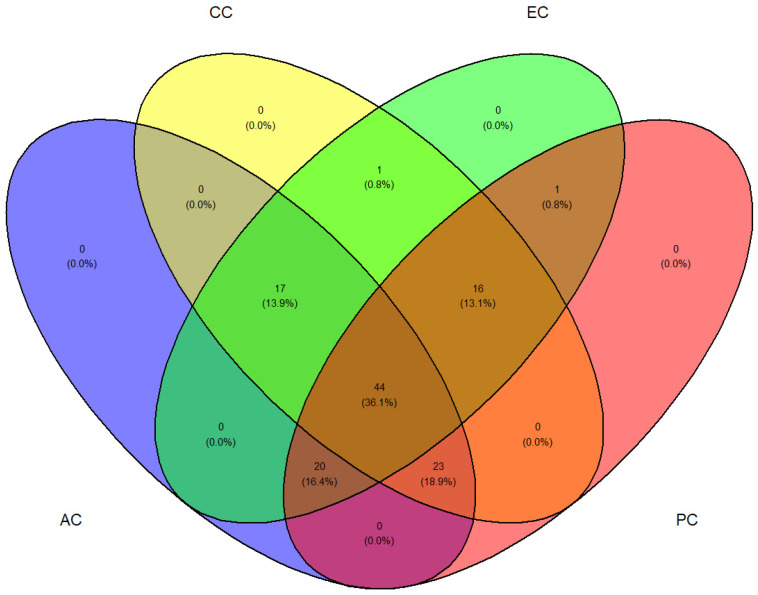
Venn diagram distribution of nutritional and phytochemical metabolites in Australian coffee (AC), Colombian coffee (CC), Ethiopian coffee (EC) and Peruvian coffee (PC).

The distribution of individual phytochemicals among coffee based on their origins was conducted through a Venn diagram in RStudio (Figure 3). Interestingly, a total of 44 (36.1%) phytochemical metabolites was observed in all coffee samples while 17 (13.9%) phytochemical metabolites overlapped in Australian, Colombian and Ethiopian coffee. A total of 16 (13.1%) phytochemical metabolites overlapped in Colombian, Ethiopian and Peruvian coffee while a total of 20 (16.4%) phytochemical metabolites over-lapped in Australian, Ethiopian and Peruvian coffee. The Venn diagram analysis gives a quick look at the distribution of phytochemicals among coffee samples.

### 2.5. Heatmap Clustering of Abundant Individual Phenolic Metabolites in Coffee Samples

**Figure 4:**
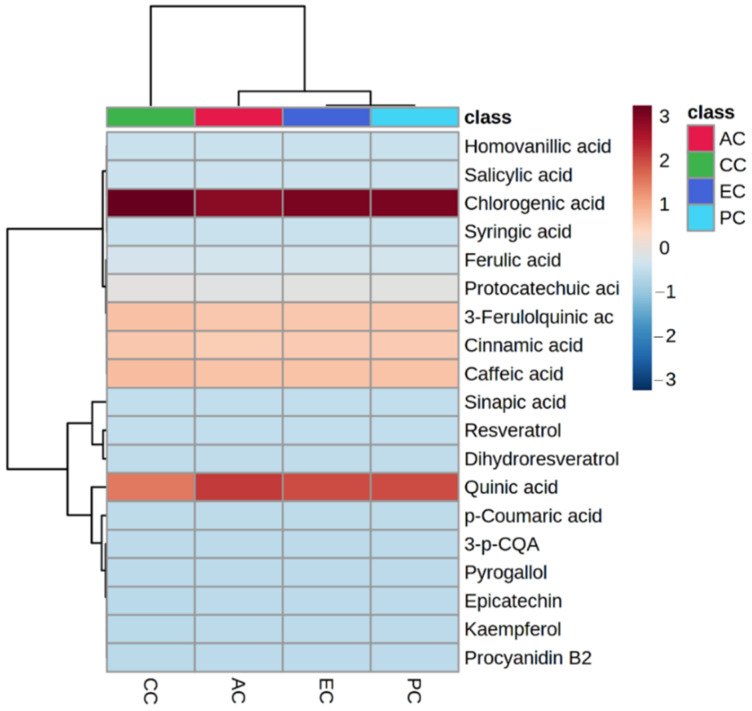
Heatmap clustering of abundant phenolic metabolites in Australian coffee (AC), Colombian coffee (CC), Ethiopian coffee (EC) and Peruvian coffee (PC).

LC-MS/MS is a frequently used analytical tool for the quantification of phenolic and metabolites in various food samples. In this study, we illustrated nineteen abundant phenolic metabolites based on their peak area in these coffee samples (Figure 4).

Chlorogenic acid and quinic acid were quantified in highest amounts, respectively. Previously, Durak et al. [50] also quantified caffeoyl quinic acid isomers from 19.8–40.7 mg/g in coffee samples through HPLC. Caffeic acid, 3-ferulolquinic acid and cinnamic were also quantified in higher amounts. Previously, Wen et al. [51] also quantified chlorogenic acid in coffee silverskin while Król, Gantner, Tatarak and Hallmann [17] quantified chlorogenic acid (5.94 mg/g) and caffeic acid (0.058 mg/g) in freshly roasted organic *C. arabica* originating from Brazil. Ferulic acid, caffeic acid, *p*-coumaric acid, caffeoylquinic acids feruloylquinic acids were reported by Ahmed Ali et al. [52]. Two hydrobenzoic-acid derivatives (salicylic acid and protocatechuic acid) were also quantified in considerable amounts in these samples, respectively. Previously, salicylic acid was quantified in the range of 0.121–0.158 mg/g in *C. arabica* [17]. Three flavonoids (epicatechin, kaempferol and PCB2 were also quantified, respectively, in studied coffee samples. Previously, Król, Gantner, Tatarak and Hallmann [17] also quantified kaempferol from 0.002 to 0.14 mg/g in different *C. arabica* samples. Two stilbenes resveratrol and dihydroresveratrol were also quantified in small amounts. Caffeine was also present in *C. arabica* in higher concentrations depicted in the peak area. The concentration of bioactive metabolites varies from sample to sample due to various geographical and roasting conditions.

### 2.6. Molecular Docking

**Figure 5:**
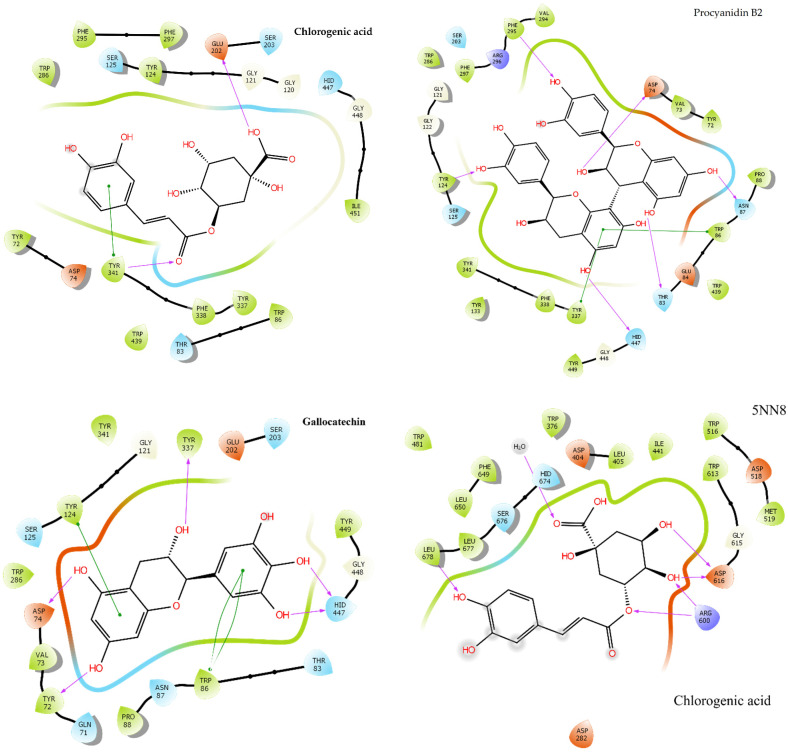

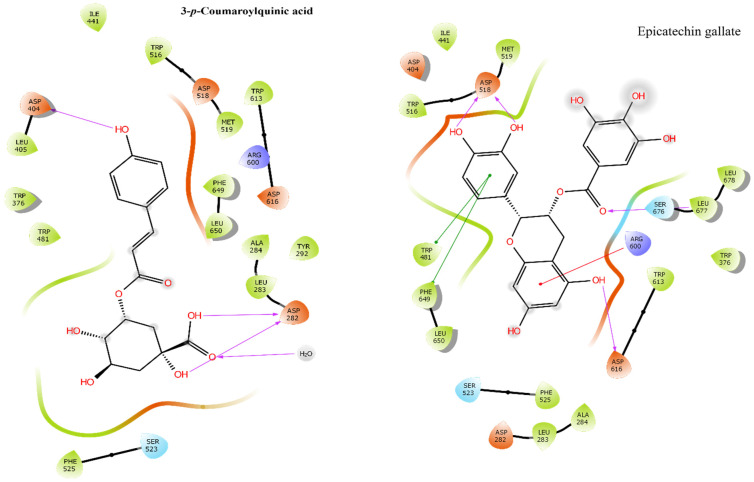
The estimated binding geometry of chlorogenic acid, procyanidin B2, gallocatechin in AChE and chlorogenic acid, 3-*p*-coumaroylquinic acid and epicatechin gallate in α-glucosidase. All hydrogen bonds are presented in pink color with single arrows while active side residues are named with three letters.

The potential of coffee bioactive metabolites towards anti-diabetic and anti-Alzheimer activities were predicted through molecular docking (Figure 5). The calculated binding and glide energies of target bioactive compounds of *C. arabica* in acetylcholinesterase (7E3I) and α-glucosidase (5NN8) are given in Table S2, while the binding geometry of chlorogenic acid, procyanidin B2 and gallocatechin in AChE while chlorogenic acid, 3-*p*-coumaroquinic acid, and epicatechin gallate in α-glucosidase (5NN8) are given in Figure 5. Chlorogenic acid in AChE (7E3I) generated hydrogen bonding with TYR 341 and GLU 202 while π−π stacking was observed with TYR 341. Procyanidin B2 in 7E3I made hydrogen bonds with ASP 74, THR 83, TYR 124, ASN 87, HID 447 and PHE 295 π−π interactions were observed with TRP 86 and TYR 337. Gallocatechin made hydrogen bonds with ASP 74, HID 447, TYR 72 and TYR 337 while double π−π interactions were observed with TRP 86. The calculated binding energy of chlorogenic acid, procyanidin B2, gallocatechin in AChE (7E3I) was −9.52, −15.26, and −11.07, respectively (Table S1). It is observed that flavonoids, including procyanidin B2, epicatechin gallate, vitexin, gallocatechin, myricetin and catechin, in *C. arabica* have higher anti-Alzheimer potential than other compounds. Interestingly, 3-*O*-sinapoylquinic acid and 3-*p*-coumaroylquinic acid have higher binding energies (−10.20 kcal/mol and −10.10 kcal/mol) than chlorogenic acid. Caffeine has the least binding energy (−5.41 kcal/mol) in 7E3I, which indicates that caffeine does not have as potent anti-Alzheimer activity as other compounds. It can be predicted that the anti-Alzheimer potential of coffee be attributed towards phenolic metabolites in coffee. Melatonin also has strong binding affinity (−9.24 kcal/mol) in 7E3I, which indicates that it also has potent anti-Alzheimer activity. In addition, chlorogenic acid in 5NN8 made hydrogen bonds with LEU 678 and one hydroxyl group, ARG 600 with the OH and O group, and ASP 616 with two hydroxyl groups. Chlorogenic acid has higher binding affinity (−8.17 kcal/mol) in 5NN8 after acarbose (−9.85 kcal/mol), which indicates that chlorogenic acid has potent anti-diabetic activity [53]. Moreover, 3-*p*-coumaroylquinic acid made two hydrogen bonds with ASP 282 and one hydrogen bond with ASP 404 and the calculated binding energy of 3-*p*-coumaroylquinic acid in 5NN8 was −7.38 kcal/mol. Epicatechin gallate made two hydrogen bonds with ASP 518 and one with ASP 616 and LEU 677 as well as π−π stacking with TRP 481 and PHE 649 and one pi-cation with ARG 600. These findings were predicted based on their binding energies and the different bondings of target compounds in 7E3I and 5NN8. It is observed that chlorogenic acid is a potent bioactive compound that mainly contributes to anti-Alzheimer’s and antidiabetic activities due it’s higher concentration in *C. arabica* and binding affinity in 7E3I and 5NN8. Molecular docking is a virtual method to predict the possible interactions between potential inhibitors and respective enzymes (AChE and α-glucosidase). Therefore, the activity of individual bioactive metabolites towards enzymes’ inhibition is important [54].

## 3. Materials and Methods

### 3.1. Materials

Analytical, HPLC, and LCMS grade chemicals were used in this study. Folin–Ciocalteu reagent, gallic acid, sodium carbonate, aluminium chloride, hydrated sodium acetate, quercetin, vanillin, sulfuric acid, l-ascorbic acid, iron(III) chloride hexahydrate (Fe(III)Cl_3_∙6H_2_O), 2,4,6-tripyridyl-*S*-triazine (TPTZ), ABTS, potassium persulfate, potassium ferrocyanide (III), trichloroacetic acid (TCA), ferric chloride, sodium phosphate dibasic heptahydrate (Na_2_HPO_4_∙7H_2_O), sodium phosphate monobasic monohydrate (Na_2_HPO_4_∙H_2_O), hydrochloric acid (HCl), ammonium molybdate, trisodium phosphate, ferrozine, ferrous chloride, iron(II) chloride, ethylene diamine tetraacetic acid (EDTA), iron(II) sulfate heptahydrate, and 3-hydroxybenzoic acid were purchased from Sigma Aldrich (Castle Hill, NSW, Australia). Hydrogen peroxide (30%) and sodium carbonate anhydrous were purchased from Chem-Supply Pty Ltd. (Adelaide, SA, Australia), and 98% sulfuric acid was purchased from RCI Labscan (Rongmuang, Thailand). HPLC and LC-MS grade reagents, including ethanol, methanol, acetonitrile, formic acid, and iron(III) chloride anhydrous, were purchased from Thermo Fisher Scientific Inc. (Scoresby, VIC, Australia).

### 3.2. Extraction and Preparation of C. arabica Samples for Analysis

The medium-roasted coffee samples of Colombian, Ethiopian and Peruvian origins were purchased from local store in Melbourne, Australia while Australian grown coffee was purchased from BunCoffee (www.buncoffee.com.au, accessed on 12 May 2022). Coffee beans were ground with laboratory grinder. The method of Solomakou et al. [20,55] were used to extract bioactive metabolites from *C. arabica* samples with modifications. Two-gram sample from each coffee was dissolved in 20 mL of 80% methanol in 1% formic acid. The extraction process was performed at 4 °C and 150 rpm for 8 h in an orbital shaker (ZWYR-240, LABWIT, Shanghai, China) and further extracted under ultrasound (Q55, Qsonica, Newton, CT, USA) with 70 amplitudes for one minute at room temperature and tubes immediately transferred into an ice box. Extractions were centrifuged at 4000 rpm for 30 min, filtered through a 0.45 μm syringe filter, and stored at −20 °C for further analysis.

### 3.3. Measurement of Total Polyphenols and Total Flavonoids

The estimation of total phenolic content was carried out by following the previously established methods [20] while all tests were performed in triplicate. Total phenolic content (TPC) and total flavonoid content (TFC) were quantified by following the methods of Sharifi-Rad et al. [56], Muhammad et al. [57], Zahid et al. [58] and Chou et al. [59] with minor modifications, as provided in the Supplementary Materials.

### 3.4. Measurement of Antioxidant Potential of C. arabica

The antioxidant activity was quantified by the free-radical scavenging effect on the DPPH radical method of Peng et al. [60] with few modifications, against a standard curve of 0–50 μg/mL ascorbic acid (C_6_H_8_O_6_). The methods of Ferreira et al. [61] and Bashmil et al. [62] after modifications were used to measure the ABTS for *C. arabica*. ^•^OH-RSA was determined by following the method of Smirnoff and Cumbes [63], with some modifications. The detailed description of all protocols is given in the Supplementary Materials.

### 3.5. LC-MS/MS Identification and Quantification of Phytochemical Metabolites

The method of Ali, Bashmil, Cottrell, Suleria and Dunshea [19] was used for the identification and complete profiling of *C. arabica* samples. The Agilent 6520 Accurate-Mass QTOF (Agilent, Santa Clara, CA, USA) equipped with Agilent HPLC 1200 series was applied in positive and negative modes with column Synergi Hydro-RP (4 μm particle size, 4.6 mm internal diameter, and 250 mm length with 80 Å pore size) and the flow rate was set at 600 μL/min. An aliquot of 20 μL from each extract was injected while gradient was 0–10 min (10–20% B), 10–20 min (20–25% B), 20–30 min (25–30% B), 30–40 min (30–45% B) 40–50 min (45–60% B), 50–60 min (60–80% B) (60–65 min (60–80% B), 65–67 min (90–100% B), 67–68 min (100–10% B) and 68–70 min (10%). Mobile phase A was 0.1% formic acid in water and mobile phase B was 0.1% formic acid in acetonitrile. A full scan mode was achieved in the range of 100–1300 *m*/*z* with the following conditions: capillary voltage (3500 V), nozzle voltage (500 V), nitrogen gas flow rate (9 L/min) at 325 °C, and nebulization was set as 45 psi while 10, 20 and 40 eV collision energies were used for the fragmentation of metabolites. Agilent MassHunter Workstation Software (Version B.06.01, Agilent, Santa Clara, CA, USA) used for data analysis. Identification and characterization of phytochemicals in coffee samples was completed following the including Personal Compounds Database and Library (PCDL) for metabolites, MassBank Europe Mass Spectral database (https://massbank.eu, accessed on 12 May 2022), MassBank (http://massbank.jp, accessed on 12 May 2022), Human Metabolome Database (https://hmdb.ca, accessed on 12 May 2022) and FooDB (https://foodb.ca, accessed on 12 May 2022). A total of 19 phenolic compounds were semi-quantified in this experiment.

### 3.6. Molecular Docking and Metabolite-Metabolite Networking of Selected Compounds

To investigate theoretically the role of abundant individual phenolic metabolites of *C. arabica* towards anti-diabetic and anti-Alzheimer potential, molecular docking was conducted by following the method of Anil et al. [64]. The structure of human lysosomal acid alpha-glucosidase in complex with acarbose (PDB ID: 5NN8) and X-ray crystallographic structure of human acetylcholinesterase in complex with tacrine (PDB ID: 7E3I) were directly obtained from the Protein Data Bank (https://www.rcsb.org, accessed on 12 May 2022) while all the structures of the selected compounds were prepared in ChemDraw (version 12.0.2, PerkinElmer, Waltham, MA, USA). Molecular docking was conducted in Maestro (version 11.2, Schrödinger, New York, NY, USA). The optimization process was completed through the addition of hydrogen atoms, completion of bond orders, deletion of water molecules, assignment of hydrogen bonds and fixed the potential of receptor atoms and energy minimization using the OPLS_2005 force field. The selected compounds were docked into the active site of α-glucosidase (5NN8) and acetylcholinesterase (7E3I).

### 3.7. Statistical Analysis

The data of the phenolic contents and the antioxidant assays were represented as the means ± standard deviation and one-way analysis of variance (ANOVA) was conducted through Minitab Program for Windows version 18.0 (Minitab, LLC, State College, PA, USA).

## 4. Conclusions

The obtained results indicate that *C. arabica* contains nutritional and phytochemical metabolites which have vital health-promoting properties. *C. arabica* contains amino acids, organic acids and fatty acids that are nutritional metabolites. In this study, we tentatively identified a total of 136 bioactive metabolites. The Colombian coffee has higher total phenolic content than other studied coffees. This depicts that total phenolic content can vary with geographical locations and roasting conditions. Phenolic compounds are secondary plant metabolites that have many health benefits. In this study, quinic acid derivatives, especially quinic acid, chlorogenic acid and 3-feruloylquinic acid, were quantified in higher amounts than other phenolic metabolites. Furthermore, this study will help to understand the role of these metabolites in the flavor and taste profiles of the coffees. Molecular docking further predicted that the anti-diabetic and anti-Alzheimer potential of coffee could be due to the phenolic metabolites in coffee rather than caffeine, which has the least binding affinity in acetylcholinesterase and α-glucosidase enzymes. Further, clinical data of individual phenolic metabolites will be required to establish the actual role of these phenolic metabolites.

## Figures and Tables

**Table 1:** Quantification of phenolic contents and their antioxidant activities.

Variables	TPC mg GAE/g	TFC mg QE/g	DPPH mg AAE/g	ABTS mg AAE/g	OH-RSA mg AAE/g
Australian Coffee	10.97 ± 0.74 ^c^	1.13 ± 0.05 ^b^	3.11 ± 0.06 ^b^	10.96 ± 0.40 ^a^	19.66 ± 0.31 ^c^
Colombian Coffee	17.74 ± 0.32 ^a^	1.36 ± 0.05 ^a^	3.61 ± 0.06 ^a^	17.17 ± 0.15 ^a^	23.62 ± 0.47 ^a^
Ethiopian Coffee	14.30 ± 0.27 ^b^	1.13 ± 0.16 ^b^	3.12 ± 0.09 ^b^	11.54 ± 0.09 ^b^	21.96 ± 0.74 ^b^
Peruvian Coffee	10.24 ± 0.73 ^c^	1.01 ± 0.13 ^bc^	1.55 ± 0.03 ^c^	6.17 ± 0.28 ^c^	17.99 ± 0.82 ^d^

Values are presented as mean ± standard deviation per gram of powder of coffee (*n* = 3). Values within same column with different superscript letters (a–d) are significantly (*p* < 0.05) different from each other. TPC (total phenolic content); TFC (total flavonoid content); 2,2′-diphenyl-1-picrylhydrazyl (DPPH), 2,2′-azino-bis-3-ethylbenzenzothiazoline-6-sulfonic acid (ABTS), and hydroxy-radical scavenging activity (^•^OH-RSA).

**Table 2:** Pearson correlation between phenolic contents and their antioxidant activities.

Variables	TPC	TFC	DPPH	ABTS
TFC	0.92 *			
DPPH	0.76	0.84		
ABTS	0.92 *	0.99 **	0.92 *	
^•^OH-RSA	0.97 **	0.90	0.87	0.94 *

** Significant correlation at *p* < 0.05, * significant correlation at *p* < 0.05.

## Data Availability

The data is available in the Supplementary Material.

## Supplementary Materials

The following supporting information can be downloaded at: https://www.mdpi.com/article/10.3390/molecules27165126/s1, Figure S1: Base Peak Chromatogram (BPC) of Australian, Colombian, Ethiopian and Peruvian coffee in positive (black color) and negative mode (blue color); Figure S2: MS/MS spectra of some selected compounds; Table S1: LC-ESI-QTOF-MS/MS identification of bioactive metabolites in *C. arabica* of different origins; Table S2: The calculated binding and glide energies of selected compounds in 7E3I and 5NN8.
