# Supplementary material for: A Comparative Study for Nutritional and Phytochemical Profiling of Coffea arabica (C. arabica) from Different Origins and Their Antioxidant Potential and Molecular Docking
Source: Molecules. 2022 Aug 11;27(16):5126. doi: 10.3390/molecules27165126 (PMC9416486; doi:10.3390/molecules27165126)
Supplement: Supplementary file 1 [file molecules-27-05126-s001.zip › molecules-1831534-supplementary.pdf]

Supplementary Materials

A Comparative Study for Nutritional and Phytochemical Profiling of *Coffea arabica* (*C. arabica*) from Different Origins and Their Antioxidant Potential and Molecular Docking

Akhtar Ali [1], Hafza Fasiha Zahid [1], Jeremy J. Cottrell [1], and Frank R. Dunshea [1,2,*]

[1] School of Agriculture and Food, Faculty of Veterinary and Agricultural Sciences, The University of Melbourne Australia, Parkville, VIC 3010, Australia;

[2] Faculty of Biological Sciences, The University of Leeds, Leeds LS2 9JT, UK

* Correspondence: fdunshea@unimelb.edu.au

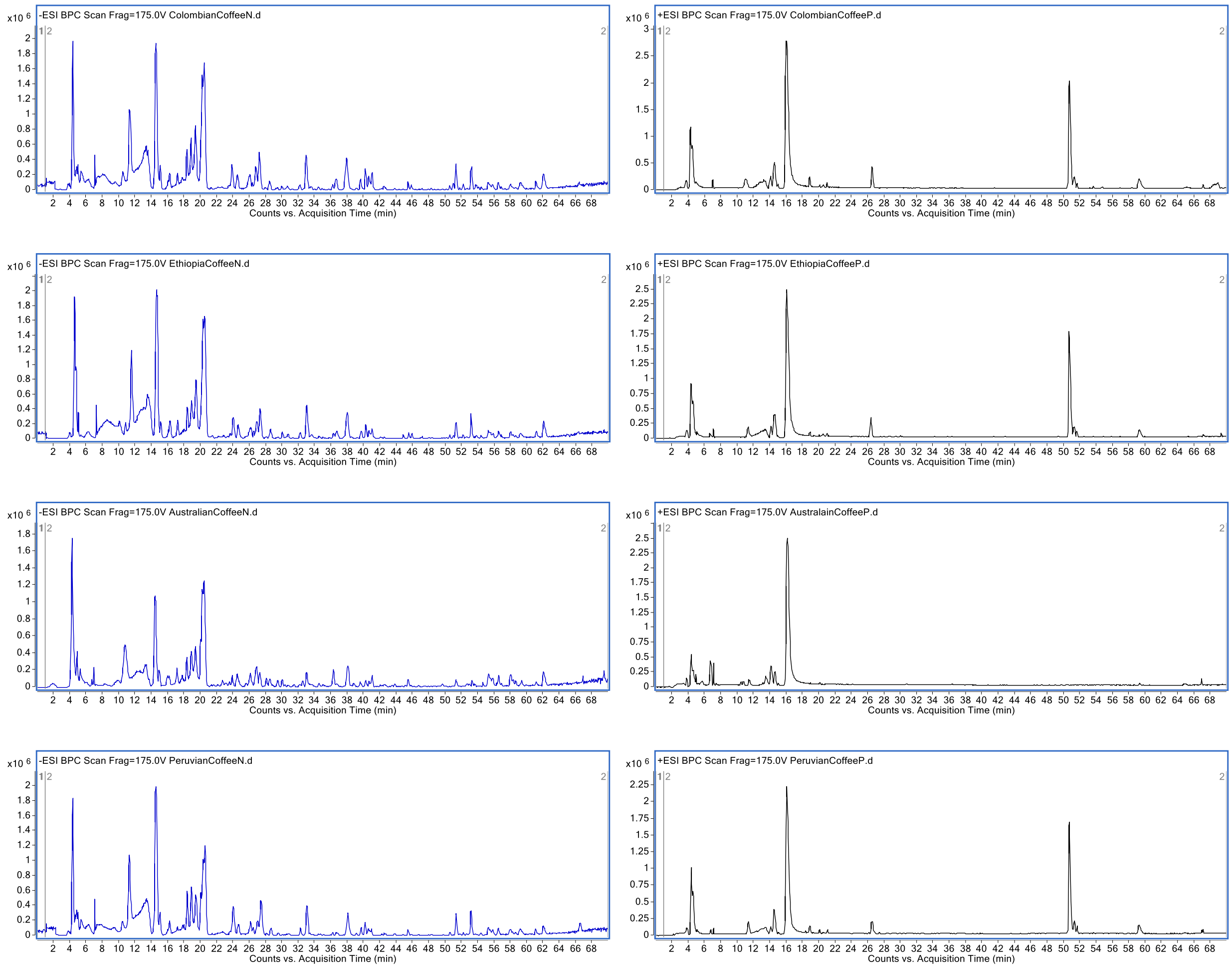

Figure S1. Base peak chromatogram (BPC) of Australian, Colombian, Ethiopian and Peruvian coffee in positive (black color) and negative mode (blue color).

Antioxidant assays

Determination of Total Polyphenols

To start, 25 μL (25% Folin–Ciocalteu reagent v/v) with 200 μL water (Milli-Q) were added to 25 μL of sample extracts in 96-well plates. Then, the plate was incubated for 5 min at 25 ◦C. Finally, 25 μL (10% v/v sodium carbonate) were added in reaction mixture and placed in the dark for 60 min at 25 ◦C and absorbance was recorded at 765 nm. The TPC was quantified by constructing the standard curve against gallic acid ranging from 0 to 200 μg/mL in ethanol. The results were documented as milligram gallic acid equivalents (GAE) per gram dry weight of samples.

Determination of Flavonoid Contents

The TFC was determined by using the AlCl3 colorimetric method. In this, 80 μL of the sample extract was mixed with 80 μL 2% aluminum chloride solution and 120 μL sodium acetate aqueous solution (50 g/L) in 96-well plates. The reaction mixture was placed in the dark at 25 ◦C for 2.5 h and absorbance was recorded at 440 nm. Measurement of all samples was made in triplicate and TFC was quantified by constructing a standard curve against 0–50 μg/mL quercetin in methanol. The results were expressed as milligram quercetin equivalents (QE) per gram dry weight of the samples (r 2 = 0.999).

DPPH Assay

To perform this, 25 μL sample extract and 275 μL 0.1 M solution of DPPH in methanol were mixed in a 96-well plate method. The reaction mixture was placed in the dark for 30 min at room temperature and absorbance was recorded at 517 nm. The anti-radical capacity of all the samples was estimated by constructing the standard curve against 0–50 μg/mL ascorbic acid in water. The results were expressed as milligram ascorbic acid equivalents per gram dry weight of the samples (mg AAE/g).

ABTS Radical Scavenging Assay

To perform this, 7 mM ABTS solution was mixed with 140 mM potassium persulfate solution. The reaction mixture was allowed to incubate in the dark for 16 h to generate an ABTS+ solution. The ABTS+ solution was diluted with ethanol to make its absorbance to 0.70 ± 0.02 at 734 nm. After this, 10 μL of sample extract was mixed with 290 μL of ABTS+ solution in a 96-well plate and allowed to incubate at 25 ◦C for 6 min and the absorbance was recorded at 734 nm. The quantification was completed by constructing the standard curve against 0–150 μg/mL of ascorbic acid in water. The results were expressed as mg AAE/g.

Hydroxyl Radical Scavenging Activity (•OH-RSA)

A 50 μL extract was mixed with 50 μL of 6 mM FeSO4·7H2O and 50 μL of 6 mM H2O2 (30%), followed by incubation at 25 ◦C for 10 min. After incubation, 50 μL of 6 mM 3-hydroxybenzoic acid was added and absorbance was measured at a wavelength of 510 nm. Ascorbic acid from 0 to 300 μg/mL was used to obtain a standard curve and data was presented in mg AAE/g.

Table S1. LC-ESI-QTOF-MS/MS identification of bioactive metabolites in *C. arabica* of different origins

No.	Proposed compounds	Molecular Formula	RT (min)	Mode of ionization	Theoretical (*m*/*z*)	Observed (*m*/*z*)	Mass Error (ppm)	MS² product ions	Samples
			Organic acids						
1	Malic acid	$C_4H_6O_5$	4.116	$[M-H]^-$	133.0142	133.0140	-1.5	73	PC, AC, CC
2	Quinic Acid	$C_7H_{12}O_6$	4.173	$[M-H]^-$	191.0561	191.0561	0.0	173, 127, 85	PC, AC, CC, EC
3	Fumaric acid	$C_4H_4O_4$	4.515	$[M-H]^-$	133.0036	115.0033	-2.6	71	AC, CC, PC
4	Citric acid	$C_6H_8O_7$	6.269	$[M-H]^-$	191.0197	191.0196	-0.5	112, 87	AC, PC, EC, CC
5	Mandelic acid	$C_8H_8O_3$	17.156	$[M-H]^-$	151.0400	151.0412	7.9	151	PC, CC
			Fatty acids						
6	Oleic acid	$C_{18}H_{34}O_2$	53.595	$[M-H]^-$	281.2485	281.2491	2.1	124	PC, AC, CC
7	Linoleic acid	$C_{18}H_{32}O_2$	58.764	$[M-H]^-$	279.2329	279.2339	3.6	204, 131	PC, AC, CC, EC
			Amino Acids						
8	L-Tyrosine	$C_9H_{11}NO_3$	4.425	$[M+H]^+$	182.0811	182.0838	17.6	167, 165, 136, 124, 122	PC, AC, CC
9	L-Pyroglutamic acid	$C_5H_7NO_3$	4.751	$[M+H]^+$	130.0498	130.0506	6.2	84	PC, AC, CC, EC
10	L-Phenylalanine	$C_9H_{11}NO_2$	5.843	$[M+H]^+$	166.0862	166.0880	10.8	151, 149, 122, 107	AC, CC, PC
			Hormones						
11	Melatonin	$C_{13}H_{16}N_2O_2$	4.820	$[M+H]^+$	233.1284	233.1309	10.7	185, 152, 93	PC, AC, CC
12	Serotonin	$C_{10}H_{12}N_2O$	4.921	$[M+H]^+$	177.1022	177.1024	0.8	177	PC, AC, CC, EC
			Alkaloids						
13	Trigonelline	C7H7NO2	4.116	$[M+H]^+$	138.0549	138.0563	10.1	120, 94, 92	EC, PC, AC, CC
14	Theophylline	$C_7H_8N_4O_2$	4.223	$[M-H]^-$	179.0574	179.0563	-6.1	164, 161, 129, 83	PC, CC, EC, AC,
15	β-Carboline	$C_{11}H_8N_2$	8.175	$[M+H]^+$	169.0760	169.0769	6.5	142, 117, 115, 70	AC, PC, CC,
16	Fontanesine B	$C_{23}H_{19}N_3O_2$	9.020	$[M+H]^+$	370.1550	370.1541	-4.1	208, 147	PC, EC, CC, AC,
17	Caffeine	$C_8H_{10}N_4O_2$	16.828	$[M+H]^+$	195.0877	195.0897	10.3	138, 110	AC, PC, CC
18	Vasicine	$C_{11}H_{12}N_2O$	31.617	$[M+H]^+$	189.1022	189.1043	11.1	171, 161, 147, 121	AC, CC, PC, EC
			Furopyrans						
19	Goniothalenol	$C_{13}H_{12}O_4$	31.521	$[M-H]^-$	231.0662	231.0664	0.9	206, 191, 177, 152, 137	EC, PC, PC, CC
			Terpenenoids						

20	Corosolic acid	$C_{30}H_{48}O_4$	55.230	$[M+H]^+$	473.3625	473.3647	4.6	418, 362	EC, PC, AC, CC
21	Kahweol	$C_{20}H_{26}O_4$	57.162	$[M+H]^+$	315.1955	315.1957	0.7	315	PC, AC, CC, EC
22	Cafestol	$C_{20}H_{28}O_3$	63.832	$[M+H]^+$	317.2111	317.2117	1.8	317	EC, CC, PC, AC,
			Carotenoids						
23	Crocetin	$C_{20}H_{24}O_4$	25.740	$[M+H]^+$	329.1747	329.1780	10.0	313, 293, 271, 183, 161	EC, CC, PC,
			Phenolic acids						
	**Hydroxybenzoic acids**								
24	*m*-Toluic acid	$C_8H_8O_2$	8.134	$[M-H]^-$	135.0451	135.0452	0.7	122, 91	EC, PC, AC, CC
25	* Protocatechuic acid	$C_7H_6O_4$	10.346	$[M-H]^-$	153.0193	153.0197	2.6	109	PC, AC, CC, EC
26	4-Hydroxybenzoic acid 4-*O*-glucoside	$C_{13}H_{16}O_8$	17.296	$[M-H]^-$	299.0772	299.0789	5.7	255, 137	EC, AC, CC, PC
27	* Syringic acid	$C_9H_{10}O_5$	22.003	$[M-H]^-$	197.0455	197.0466	5.6	182, 153, 138, 123	EC, AC, PC, CC
28	* 2-Hydroxybenzoic acid	$C_7H_6O_3$	28.002	$[M-H]^-$	137.0244	137.0252	5.8	93	EC, PC, CC, AC
	**Hydroxycinnamic acids**								
29	*p*-Coumaric acid 4-*O*-glucoside	$C_{15}H_{18}O_8$	10.391	$[M-H]^-$	325.0929	325.0937	2.5	163	PC, AC, CC, EC
30	Methyl chlorogenate	$C_{16}H_{18}O_9$	12.624	$[M+H]^+$	369.1180	369.1213	8.9	177, 145	EC, AC, CC, PC
31	Dihydroferulic acid	$C_{10}H_{12}O_4$	14.611	$[M-H]^-$	195.0663	195.0664	0.5	151, 135	AC, PC, EC, CC
32	* 3-Caffeoylquinic acid	$C_{16}H_{18}O_9$	14.752	**$[M-H]^-$	353.0878	353.0878	0.0	191, 179, 161, 135	PC, AC, CC
33	* Caffeic acid	$C_9H_8O_4$	15.013	$[M-H]^-$	179.035	179.0367	9.5	161, 135	EC, AC, PC, CC
34	1-Sinapoyl-2-feruloylgentiobiose	$C_{33}H_{40}O_{18}$	15.290	$[M-H]^-$	723.2142	723.2084	-8.0	529, 449	PC, AC, CC
35	* Rosmarinic acid	$C_{18}H_{16}O_8$	15.876	$[M-H]^-$	359.0772	359.0791	5.3	197, 179, 161, 135	PC, AC, CC, EC
36	3-*p*-Coumaroylquinic acid	$C_{16}H_{18}O_8$	17.558	$[M-H]^-$	337.0929	337.0924	-1.5	191, 173, 93	AC, CC, PC
37	Feruloyl glucose	$C_{16}H_{20}O_9$	18.655	$[M-H]^-$	355.1034	355.103	-1.1	177, 149, 133, 59	AC, PC, EC, CC
38	1,2-Diferuloylgentiobiose	$C_{32}H_{38}O_{17}$	18.685	**$[M-H]^-$	693.2036	693.203	-0.9	499, 191	AC, PC, CC
39	3-Sinapoylquinic acid	$C_{18}H_{22}O_{10}$	18.722	$[M-H]^-$	397.114	397.1128	-3.0	353, 233, 191	PC, AC, CC
40	3-Feruloylquinic acid	$C_{17}H_{20}O_9$	18.983	** $[M-H]^-$	367.1034	367.1034	0.0	193, 191, 173, 161	PC, AC, CC, EC
41	* Cinnamic acid	$C_9H_8O_2$	19.515	$[M-H]^-$	147.0451	147.0457	4.1	103	EC, AC, CC, PC
42	1,5-Dicaffeoylquinic acid	$C_{25}H_{24}O_{12}$	20.336	$[M-H]^-$	515.1195	515.1179	-3.1	353, 191, 179, 161	AC, PC, EC, CC
43	1-*O*-Sinapoyl-beta-D-glucose	$C_{17}H_{22}O_{10}$	20.384	$[M-H]^-$	385.114	385.1132	-2.1	367, 223, 205	PC, CC, AC
44	*p*-Coumaroyl glycolic acid	$C_{11}H_{10}O_5$	21.250	$[M-H]^-$	221.0455	221.0471	7.2	177, 119, 65	PC, AC, CC
45	* Ferulic acid	$C_{10}H_{10}O_4$	22.340	$[M-H]^-$	193.0506	193.0507	0.5	178, 149, 134	PC, AC, CC, EC
46	1-Sinapoyl-2,2′-diferuloylgentiobiose	$C_{43}H_{48}O_{21}$	23.155	$[M-H]^-$	899.2615	899.2533	-9.1	675, 193	AC, CC, PC

47	2-Feruloyl-1,2′-disinapoylgentiobiose	$C_{44}H_{50}O_{22}$	23.74	$[M-H]^-$	929.2721	929.2707	-1.5	223, 205, 193	AC, PC, EC, CC
48	* Sinapic acid	$C_{11}H_{12}O_5$	24.43	$[M-H]^-$	223.0612	223.0607	-2.2	205, 163	AC, EC, PC, CC
49	1-Caffeoyl-5-feruloylquinic acid	$C_{26}H_{26}O_{12}$	25.86	$[M-H]^-$	529.1351	529.1324	-5.1	353, 179, 161, 135	PC, AC, CC
50	1,2,2′-Triferuloylgentiobiose	$C_{42}H_{46}O_{20}$	27.446	$[M-H]^-$	869.2509	869.25	-1.0	675, 193	PC, AC, CC, EC
51	Cinnamoyl glucose	$C_{15}H_{18}O_7$	27.504	$[M-H]^-$	309.0979	309.1008	9.4	147, 131, 103	AC, CC, PC
52	* *p*-Coumaric acid	$C_9H_8O_3$	27.561	$[M-H]^-$	163.04	163.0396	-2.5	109	AC, PC, EC, CC
53	3,5-Diferuloylquinic acid	$C_{27}H_{28}O_{12}$	27.896	$[M-H]^-$	543.1508	543.148	-5.2	367, 191	PC, CC, AC
54	*p*-Coumaric acid ethyl ester	$C_{11}H_{12}O_3$	28.32	$[M+H]^+$	193.0859	193.0859	0.0	191, 145	PC, AC, CC
55	Verbascoside A	$C_{31}H_{40}O_{16}$	28.517	$[M-H]^-$	667.2243	667.2261	2.7	283, 94	PC, AC, CC, EC
			Other phenolic acids						
	Hydroxyphenylacetic acids								
56	2-Hydroxy-2-phenylacetic acid	$C_8H_8O_3$	23.316	$[M-H]^-$	151.04	151.0405	3.3	136, 92	PC, AC, CC
	3,4-Dihydroxyphenylacetic acid	$C_8H_8O_4$	25.666	$[M-H]^-$	167.035	167.0346	-2.4	149, 123	PC, AC, CC, EC
57	* Homovanillic acid	$C_9H_{10}O_4$	26.579	$[M-H]^-$	181.0506	181.0496	-5.5	137	PC, AC, CC
58	Hydroxyphenylpentanoic acids								PC, AC, CC, EC
59	4-Hydroxy-(3′,4′-dihydroxyphenyl)valeric acid	$C_{11}H_{14}O_5$	16.585	$[M-H]^-$	225.0768	225.0755	-5.8	207, 163, 123	AC, CC, PC
	Hydroxyphenylpropanoic acids								AC, PC, EC, CC
60	3-Phenylpropionic acid	$C_9H_{10}O_2$	23.155	$[M-H]^-$	149.0608	149.0598	-6.7	105	PC, CC
61	3-(3,4-Dihydroxyphenyl)-2-methoxypropionic acid	$C_{10}H_{12}O_5$	23.35	$[M-H]^-$	211.0612	211.061	-0.9	193, 167, 123	PC, AC, CC
			Flavonoids						
	**Flavanols**								
62	3′-*O*-Methyl-(-)-epicatechin-7-*O*-glucuronide	$C_{22}H_{24}O_{12}$	4.94	$[M-H]^-$	479.1195	479.1194	-0.2	149, 121	PC, AC, CC, EC
63	(+)-Gallocatechin 3-*O*-gallate	$C_{22}H_{18}O_{11}$	6.905	$[M+H]^+$	459.0922	459.0928	1.3	289	PC, AC, CC, EC
64	* (-)-Epicatechin	$C_{15}H_{14}O_6$	9.804	$[M-H]^-$	289.0717	289.0713	-1.4	245	EC, AC, CC, PC
65	* Procyanidin dimer B2	$C_{30}H_{26}O_{12}$	23.061	$[M-H]^-$	577.1351	577.1398	8.1	451, 425	AC, PC, EC, CC
66	Epicatechin gallate	$C_{22}H_{18}O_{10}$	24.073	$[M-H]^-$	441.0827	441.0856	6.6	289	EC, PC, CC, AC
67	Gallocatechin	$C_{15}H_{14}O_7$	26.235	$[M-H]^-$	305.0667	305.0687	6.7	245	AC, PC, AC, CC
68	3′-*O*-Methylepicatechin	$C_{16}H_{16}O_6$	28.75	$[M-H]^-$	303.0874	303.0897	7.6	271, 163	PC, AC, CC, EC
	**Flavonols**								AC, CC, PC

69	Kaempferol 7-*O*-glucoside	$C_{21}H_{19}O_{11}$	3.369	$[M-H]^-$	446.0854	446.0873	4.3	285	AC, PC, EC, CC
70	Jaceidin 4′-*O*-glucuronide	$C_{24}H_{24}O_{14}$	4.141	$[M+H]^+$	537.1239	537.1257	3.4	361	PC, CC
71	Myricetin 3-*O*-rutinoside	$C_{27}H_{30}O_{17}$	4.141	**$[M+H]^+$	627.1556	627.1604	7.7	319	PC, AC, CC
72	Isorhamnetin	$C_{16}H_{12}O_7$	4.669	$[M+H]^+$	317.0656	317.068	7.6	302, 229, 152	PC, AC, CC, EC
73	Myricetin 3-*O*-rhamnoside	$C_{21}H_{20}O_{12}$	5.787	[M+H]+	465.1028	465.1056	6.0	319, 301	AC, CC, PC
74	Kaempferol-3-*O*-(2′′-rhamnosyl-galactoside) 7-*O*-rhamnoside	$C_{33}H_{40}O_{19}$	26.665	$[M-H]^-$	739.2091	739.2052	-5.3	575, 431, 163	AC, PC, EC, CC
75	Kaempferol	$C_{15}H_{10}O_6$	34.542	$[M-H]^-$	285.0404	285.0423	6.5	267, 151	PC, CC
76	Quercetin 3-*O*-(6′′-acetyl-galactoside) 7-O-rhamnoside	$C_{29}H_{32}O_{17}$	59.628	$[M+H]^+$	653.1713	653.1761	7.3	489, 449, 431, 301, 285	PC, AC, CC
	**Flavones**								PC, AC, CC, EC
77	Tricin 7-neohesperidoside	$C_{29}H_{34}O_{16}$	4.715	$[M-H]^-$	637.1774	637.174	-5.3	491, 329	AC, CC, PC
78	Nobiletin	$C_{21}H_{22}O_8$	25.096	$[M-H]^-$	401.1242	401.1247	1.2	237, 188, 145, 59	AC, PC, EC, CC
79	Apigenin 6,8-di-C-glucoside	$C_{27}H_{30}O_{15}$	47.368	$[M+H]^+$	595.1658	595.171	8.7	577, 383	PC, CC
	**Flavanones**								PC, AC, CC
80	Didymin	$C_{28}H_{34}O_{14}$	4.306	$[M-H]^-$	593.1876	593.1865	-1.9	431, 285	PC, AC, CC, EC
81	Hesperetin 3′-*O*-glucuronide	$C_{22}H_{22}O_{12}$	4.635	$[M+H]^+$	479.1184	479.1204	4.2	303, 285, 177, 151	AC, CC, PC
82	Naringenin 7-*O*-glucoside	$C_{21}H_{22}O_{10}$	22.752	$[M-H]^-$	433.114	433.1129	-2.5	271, 151	AC, PC, EC, CC
	**Isoflavonoids**								
83	6′′-*O*-Malonyldaidzin	$C_{24}H_{22}O_{12}$	3.663	$[M-H]^-$	501.1038	501.1056	3.6	253	PC, AC, CC
84	3′-*O*-Methylviolanone	$C_{18}H_{18}O_6$	4.25	$[M-H]^-$	329.103	329.1001	-8.8	285, 163	PC, AC, CC, EC
85	6′′-*O*-Acetylglycitin	$C_{24}H_{24}O_{11}$	5.285	$[M-H]^-$	487.1246	487.1209	-7.6	283, 267, 59	AC, CC, PC
86	4′-Methoxy-2′,3,7-trihydroxyisoflavanone	$C_{16}H_{14}O_6$	6.488	$[M-H]^-$	301.0717	301.0725	2.7	283, 177, 135	AC, PC, EC, CC
87	Violanone	$C_{17}H_{16}O_6$	8.956	$[M-H]^-$	315.0874	315.086	-4.4	300, 285, 135	PC, CC, EC, AC
88	3′,4′,5,7-Tetrahydroxyisoflavanone	$C_{15}H_{12}O_6$	18.008	$[M-H]^-$	287.0561	287.057	3.1	269, 179	PC, AC, CC
89	Equol 7-*O*-glucuronide	$C_{21}H_{22}O_9$	21.038	$[M-H]^-$	417.1191	417.1207	3.8	399, 241	PC, AC, CC, EC
90	Glycitin	$C_{22}H_{22}O_{10}$	23.403	$[M-H]^-$	445.114	445.1166	5.8	427, 311, 267	AC, CC, PC
91	3′,4′,7-Trihydroxyisoflavanone	$C_{15}H_{12}O_5$	23.494	$[M-H]^-$	271.0612	271.0631	7.0	163, 135, 109	AC, PC, EC, CC
92	Glycitein 7-*O*-glucuronide	$C_{22}H_{20}O_{11}$	23.669	$[M-H]^-$	459.0933	459.0934	0.2	441, 283, 267	PC, AC, CC, EC
93	Dihydrobiochanin A	$C_{16}H_{14}O_5$	27.03	$[M-H]^-$	285.0768	285.0771	1.1	269, 203, 175	PC, AC, CC
94	3′-*O*-Methylequol	$C_{16}H_{16}O_4$	27.479	$[M+H]^+$	273.1122	273.112	-0.7	255, 149, 121	PC, AC, EC, CC,
95	Daidzin	$C_{21}H_{20}O_9$	29.599	$[M-H]^-$	415.1034	415.1038	1.0	253	EC, AC, CC, PC

96	Formononetin	$C_{16}H_{14}O_4$	34.046	$[M-H]^-$	269.0819	269.0824	1.9	251, 223	AC, PC, EC, CC
97	6′-Hydroxyangolensin	$C_{16}H_{16}O_5$	34.255	$[M-H]^-$	287.0925	287.0917	-2.8	181, 125	PC, CC
	**Dihydroflavonols**								PC, AC, CC
99	Dihydromyricetin 3-*O*-rhamnoside	$C_{21}H_{22}O_{12}$	4.141	$[M+H]^+$	467.1184	467.1215	6.6	321, 153	PC, AC, CC, EC
100	Dihydrochalcones								AC, CC, PC
101	3-Hydroxyphloretin 2′-*O*-xylosyl-glucoside	$C_{26}H_{32}O_{15}$	12.086	$[M-H]^-$	583.1668	583.1684	2.7	565, 289, 271	AC, PC, EC, CC
102	3-Hydroxyphloretin 2′-*O*-glucoside	$C_{21}H_{24}O_{11}$	13.591	$[M-H]^-$	451.1246	451.1252	1.3	433, 289	PC, CC
103	Phloridzin	$C_{21}H_{24}O_{10}$	25.198	$[M-H]^-$	435.1297	435.1266	-7.1	273, 255	PC, AC, CC
104	Phloretin 2′-*O*-xylosyl-glucoside	$C_{26}H_{32}O_{14}$	29.198	$[M-H]^-$	567.1719	567.1724	0.9	273, 149	PC, AC, CC, EC
	Stilbenes								
105	3′-Hydroxy-3,4,5,4′-tetramethoxystilbene	$C_{17}H_{18}O_5$	16.585	$[M-H]^-$	301.1081	301.1102	7.0	283, 255	AC, PC, EC, CC
106	Piceatannol	$C_{14}H_{12}O_4$	25.917	$[M-H]^-$	243.0663	243.0664	0.4	225, 201	PC, CC, AC, EC
107	* Resveratrol	$C_{14}H_{12}O_3$	27.715	$[M-H]^-$	227.0713	227.0705	-3.5	185, 143	PC, AC, CC
108	Piceatannol 3-*O*-glucoside	$C_{20}H_{22}O_9$	29.932	$[M-H]^-$	405.1191	405.1191	0.0	243, 225	PC, AC, CC, EC
109	Dihydroresveratrol	$C_{14}H_{14}O_3$	37.135	$[M-H]^-$	229.087	229.0864	-2.6	123, 81	AC, CC, PC
	Lignans								
110	Schisandrol B	$C_{23}H_{28}O_7$	20.491	$[M+H]^+$	417.1908	417.192	2.9	224, 193, 165	PC, CC
111	Guaiacin	$C_{20}H_{24}O_4$	20.910	$[M+H]^+$	329.1747	329.1790	13.1	313, 137	PC, AC, CC
112	7-Oxomatairesinol	$C_{20}H_{20}O_7$	35.806	$[M-H]^-$	371.1136	371.1118	-4.9	355, 221	PC, AC, CC, EC
	Other polyphenols								
	Alkylphenols								AC, PC, EC, CC
113	4-Ethylphenol	$C_8H_{10}O$	15.948	$[M+H]^+$	123.0805	123.0794	-8.9	107, 91, 77	PC, EC, AC, CC
114	3-Methylcatechol	$C_7H_8O_2$	21.711	$[M-H]^-$	123.0451	123.045	-0.8	105	PC, AC, CC
115	4-Vinylphenol	C8H8O	22.203	$[M-H]^-$	119.0502	119.0503	0.8	101, 91, 65	PC, AC, CC, EC
116	5-Pentacosylresorcinol	$C_{31}H_{56}O_2$	48.713	$[M+H]^+$	461.4353	461.4324	-6.3	443, 351	AC, CC, PC
	Hydroxybenzaldehydes								
117	*p*-Anisaldehyde	$C_8H_8O_2$	20.384	$[M-H]^-$	135.0451	135.0454	2.2	119	PC, CC
	Cinnamonaldehydes								
118	*p*-Coumaraldehyde	$C_9H_8O_2$	19.593	$[M+H]^+$	149.0597	149.0612	11.7	131, 121, 77, 65	PC, AC, CC, EC
	Coumarins								
119	Mellein	$C_{10}H_{10}O_3$	20.541	$[M-H]^-$	177.0557	177.0558	0.6	133	AC, PC, EC, CC

120	Umbelliferone	$C_9H_6O_3$	22.145	[M−H]$^-$	161.0244	161.0245	0.6	133, 117	AC, EC, PC, CC
121	4-Methylumbelliferone	$C_{10}H_8O_3$	23.341	[M−H]$^-$	175.0400	175.0396	-3.4	161, 160, 121	PC, AC, CC
122	6,8-Dimethyl-4-hydroxycoumarin	$C_{11}H_{10}O_3$	30.811	[M−H]$^-$	189.0556	189.0543	-6.9	173, 161, 149, 121, 93	PC, AC, CC, EC
123	7-(Dimethylamino)-4-methylcoumarin	$C_{12}H_{13}NO_2$	18.279	[M+H]$^+$	204.1019	204.1018	-0.5	189, 176, 160, 122	AC, CC, PC
124	Coumarin	$C_9H_6O_2$	23.155	**[M+H]$^+$	147.0295	147.0296	0.7	119, 103, 91, 65	AC, PC, EC, CC
125	Urolithin B	$C_{13}H_8O_3$	31.052	[M−H]$^-$	211.0400	211.0399	-0.5	167	PC, CC
126	5-Methoxyfuranocoumarin	$C_{12}H_8O_4$	54.83	[M−H]$^-$	215.0350	215.0339	-5.1	171	PC, AC, CC
			Tyrosols						
127	*p*-HPEA-EDA	$C_{17}H_{20}O_5$	4.141	[M+H]$^+$	305.1384	305.1378	-2.0	287, 167, 121	AC, CC, PC
128	Hydroxytyrosol	$C_8H_{10}O_3$	22.684	[M−H]$^-$	153.0557	153.0555	-1.3	123, 109	AC, PC, EC, CC
129	Hydroxytyrosol 4-*O*-glucoside	$C_{14}H_{20}O_8$	28.715	** [M+H]$^+$	317.1231	317.1228	-0.9	155, 137	PC, CC, EC, AC
			Phenolic terpenes						
130	Carnosol	$C_{20}H_{26}O_4$	32.131	[M−H]$^-$	329.1758	329.1733	-7.6	285	PC, AC, CC, EC
131	Carnosic acid	$C_{20}H_{28}O_4$	40.483	[M−H]$^-$	331.1915	331.1919	1.2	287	EC, AC, CC, PC
132	Carvacrol	$C_{10}H_{14}O$	49.831	[M−H]$^-$	149.0972	149.0969	-2.0	133	AC, PC, EC, CC
			Xanthones						
133	Mangiferin	$C_{19}H_{18}O_{11}$	4.142	[M−H]$^-$	421.0776	421.0731	-10.7	301	PC, EC, AC, CC
			Other polyphenols						
134	Phlorin	$C_{12}H_{16}O_8$	4.715	[M−H]$^-$	287.0772	287.0782	3.5	125	EC, AC, CC, PC
135	Pyrogallol	$C_6H_6O_3$	9.088	[M−H]$^-$	125.0244	125.0252	6.4	107, 97, 79	AC, PC, EC, CC
	Uncategorized								AC, EC, CC, PC,
136	Caffeoylcholine	$C_{14}H_{20}NO_4^+$	13.917	[M]$^+$	266.1392	266.1411	7.1	207, 163	EC, PC, AC, CC

Compound 32

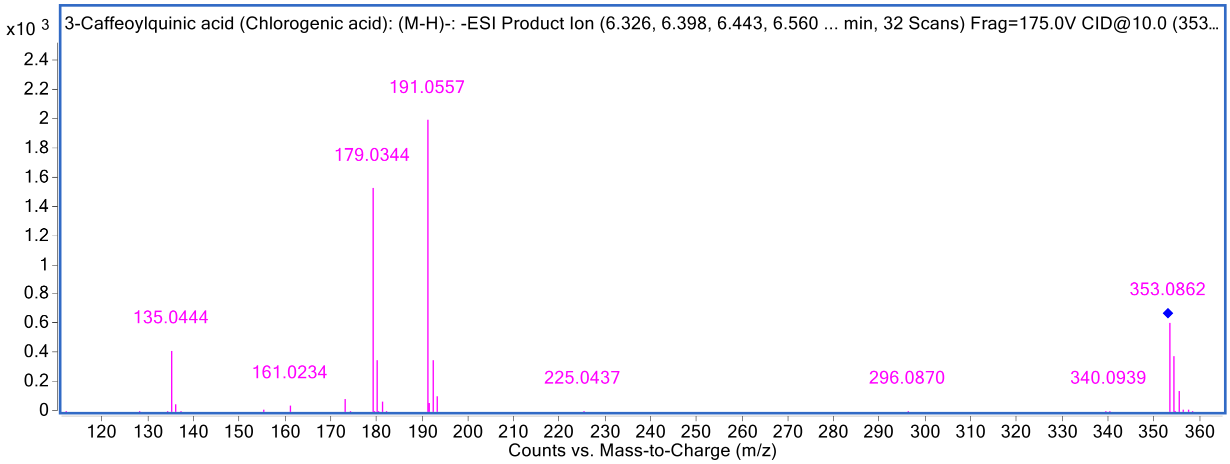

Compound 33

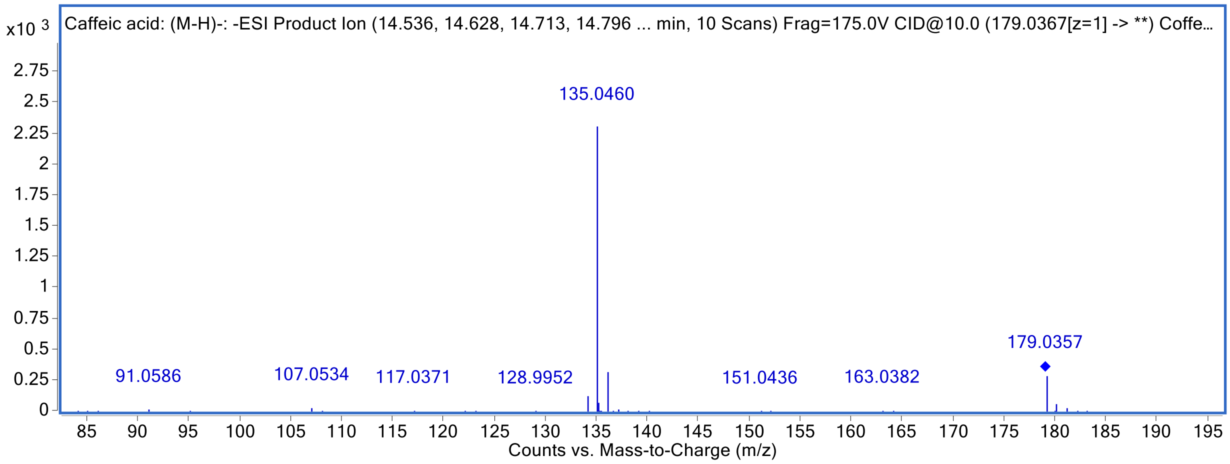

Compound 45

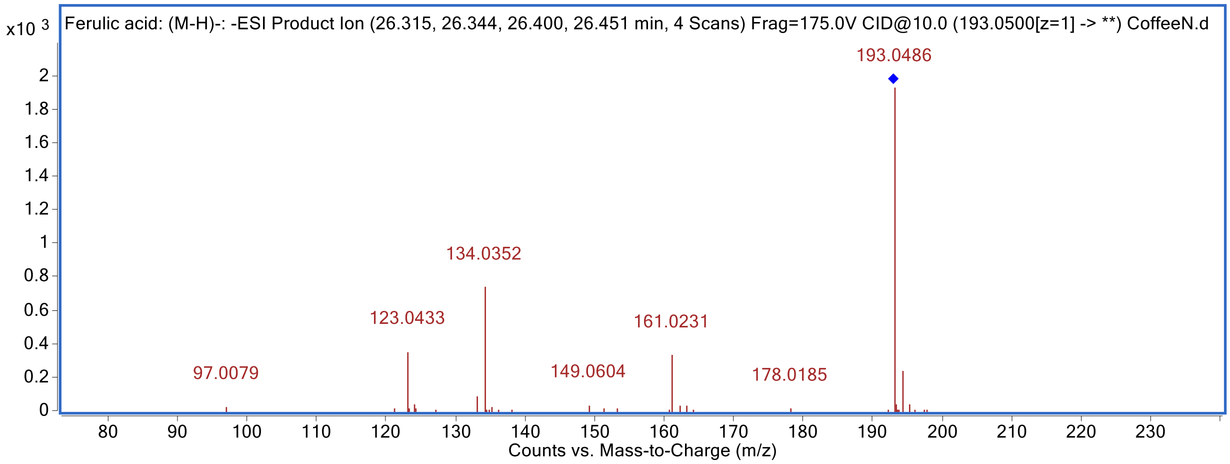

Compound 117

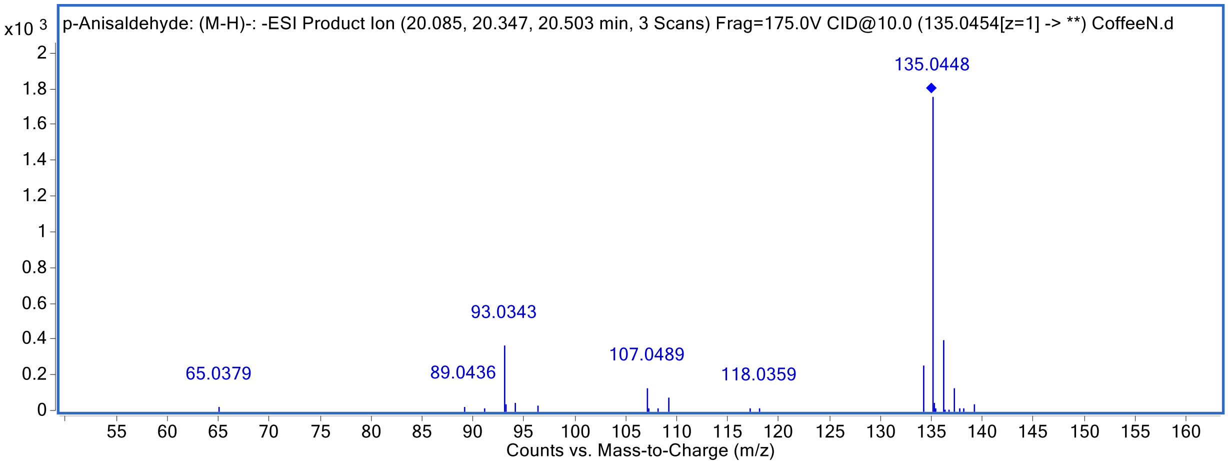

Compound 119

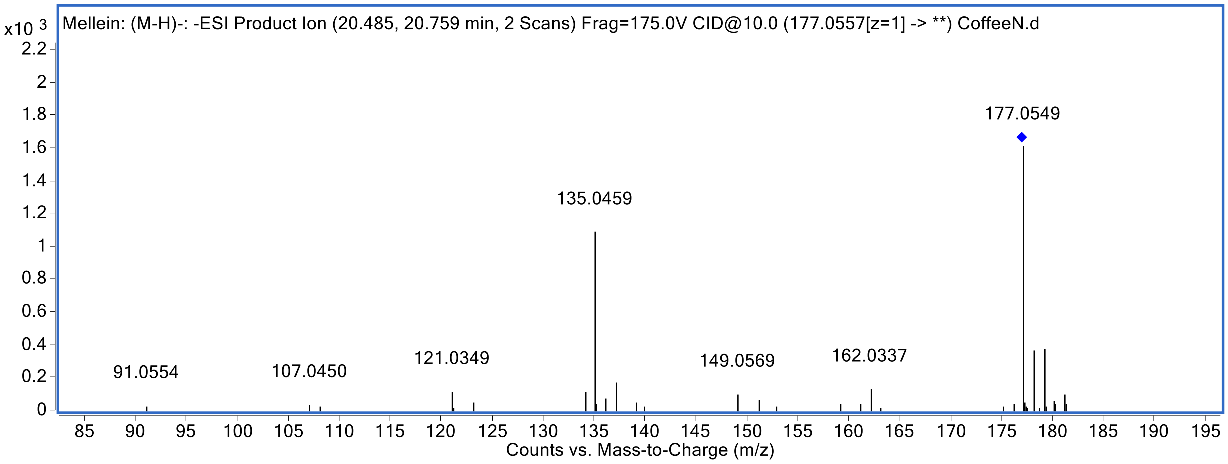

Compound 135

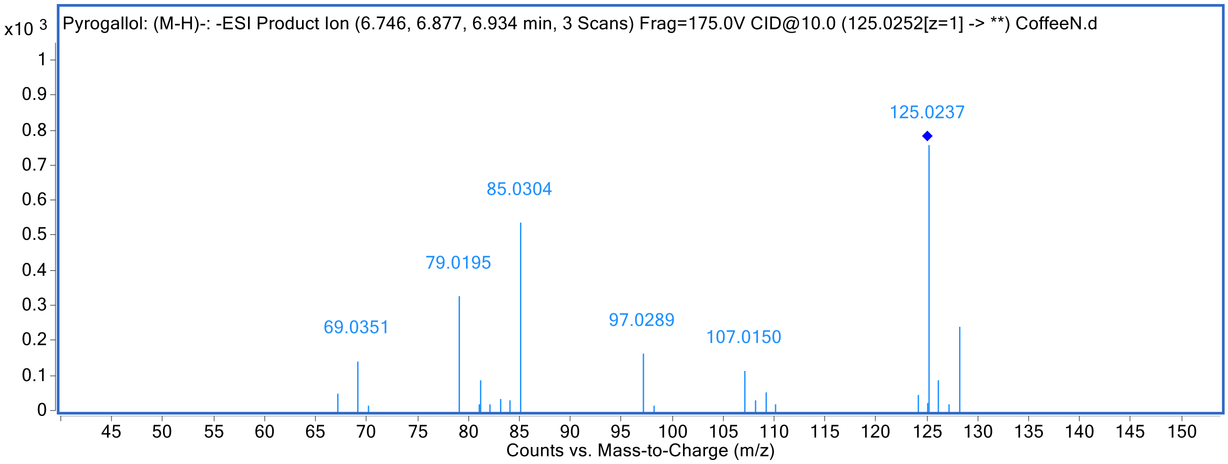

Figure S2. MS/MS spectra of some selected compounds

Table S2. The calculated binding and glide energies of selected compounds in 7E3I and 5NN8

No	Compounds in 7E3I	Binding energy (kcal/mol)	Glide energy (kcal/mol)	Compounds in 5NN8	Binding energy (kcal/mol)	Glide energy (kcal/mol)
1	Procyanidin B2	-15.26	-59.96	Acarbose (Standard)	-9.85	-58.26
2	(-)-Epicatechin gallate	-14.43	-61.84	Chlorogenic acid	-8.17	-48.67
3	Apigenin 8-C-glucoside	-12.16	-60.53	(-)-Epicatechin gallate	-7.84	-47.94
4	Dihydromyricetin	-11.19	-50.07	Naringin	-7.58	-55.25
5	(+)-Gallocatechin	-11.07	-52.41	Procyanidin B2	-7.50	-51.44
6	Myricetin	-10.84	-50.04	3-*p*-Coumaroylquinic acid	-7.38	-46.44
7	Dihydroquercetin	-10.51	-48.52	Myricetin	-6.92	-43.43
8	Dihydrorobinetin	-10.40	-46.98	Dihydromyricetin	-6.88	-43.38
9	3-*O*-Sinapoylquinic acid	-10.20	-46.90	Dihydroquercetin	-6.50	-38.83
10	3-*p*-Coumaroylquinic acid	-10.10	-44.69	Apigenin 8-C-glucoside	-6.44	-40.31
11	Luteolin	-9.93	-45.97	Dihydrorobinetin	-6.37	-42.45
12	Naringenin	-9.82	-51.78	Quinic acid	-6.30	-39.01
13	(-)-Epicatechin	-9.72	-45.12	(+)-Gallocatechin	-6.26	-37.10
14	Chlorogenic acid	-9.52	-57.79	Quinic acid	-6.09	-35.08
15	Kaempferol	-9.52	-45.45	3-*O*-Sinapoylquinic acid	-6.08	-40.79
16	Melatonin	-9.24	-42.67	(-)-Epicatechin	-6.08	-36.09
17	Carnosic acid	-8.89	-32.29	Kaempferol	-6.01	-33.74
18	Dalbergin	-8.76	-36.55	Carnosic acid	-5.91	-32.63
19	Chrysin	-8.62	-45.44	Luteolin	-5.67	-36.15
20	Dihydroresveratrol	-8.56	-36.94	Gallic acid	-5.10	-30.17
21	Isorhamnetin	-8.22	-43.44	Naringenin	-5.03	-30.66
22	Protocatechuic acid	-8.01	-30.14	Protocatechuic acid	-4.96	-29.81
23	Gallic acid	-7.97	-30.56	Isorhamnetin	-4.89	-34.55
24	3-*O*-Methylviolanone	-7.94	-39.49	Caffeic acid	-4.87	-30.58
25	Galantamine (Standard)	-7.64	-34.89	Pyrogallol	-4.80	-23.12
26	Tacrine (Standard)	-7.34	-34.38	Salicylic acid	-4.76	-22.45
27	Resveratrol	-7.33	-39.70	Syringic acid	-4.70	-30.51
28	Caffeic acid	-7.33	-38.00	Dihydroresveratrol	-4.62	-31.66
29	Vanillic acid	-7.19	-31.22	*p*-Hydroxybenzoic acid	-4.45	-28.34
30	Ferulic acid	-7.12	-42.61	Vanillic acid	-4.21	-28.96
31	4-Aminobenzoic acid	-6.69	-26.72	Coumarin	-4.13	-16.42
32	Quinic acid	-6.65	-39.11	Chrysin	-4.10	-33.75
33	*p*-Hydroxybenzoic acid	-6.64	-27.80	Melatonin	-3.94	-31.85
34	*m*-Toluic acid	-6.57	-23.37	Sinapic acid	-3.94	-27.92
35	Coumarin	-6.57	-24.00	Ferulic acid	-3.77	-26.03
36	Quinic acid	-6.55	-37.71	*m*-Toluic acid	-3.64	-23.05
37	Pyrogallol	-6.52	-24.98	Dalbergin	-3.62	-29.67
38	Syringic acid	-6.49	-32.04	Resveratrol	-3.57	-35.08
39	Salicylic acid	-6.45	-27.08	4-Aminobenzoic acid	-3.54	-23.33
40	Sinapic acid	-6.33	-41.57	3-*O*-Methylviolanone	-3.49	-36.77
41	*p*-coumaric acid	-6.27	-39.54	Cinnamic acid	-2.89	-26.12

42	Cinnamic acid	-6.25	-23.78	4-Nitrophenol	-2.42	-21.09
43	Caffeine	-5.41	-33.72	Caffeine	-2.29	-21.85
44	4-Nitrophenol	-4.30	-26.71			
